# Tweaking Microtubules to Treat Scleroderma

**DOI:** 10.1371/journal.pmed.0020415

**Published:** 2005-12-27

**Authors:** Jacob M van Laar, Tom W. J Huizinga

## Abstract

van Laar and Huizinga discuss a new study of a mouse model of scleroderma, which showed that stabilizing microtubules with paclitaxel led to reduced fibrosis.

Systemic sclerosis (SSc; also referred to as “scleroderma”) is a rare but debilitating autoimmune disease clinically characterized by skin thickening and signs and symptoms of vasculopathy, which can involve the heart, lungs, kidneys, and gut. The disease spectrum can range from limited to diffuse disease, depending on the distribution of skin involvement, specificity of autoantibodies, and type of organ involvement. Patients with extensive skin thickening and organ dysfunction in particular are at risk of premature mortality [1].

The disease poses a challenge for the treating clinician, as no proven therapy exists that improves outcome, although recent data indicate that cyclophosphamide-based regimens may be effective in a subset of patients with early disease [2]. The etiology of SSc remains enigmatic, and few genetic and environmental predisposing factors have been identified.

## Pathogenesis of SSc

**Figure 1:**
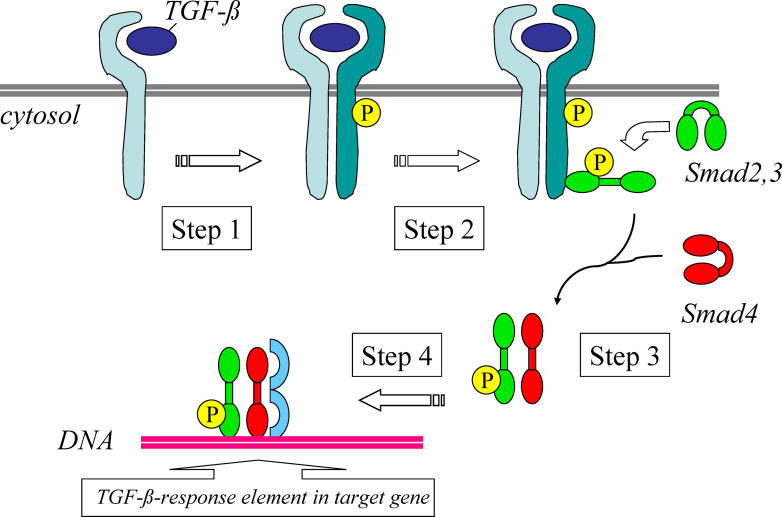
Simplified Model for Smad-Dependent Signaling Pathway Activated by TGFβ Showing the Consecutive Steps following TGFβ Binding to the Type II TGFβ receptor Step 1: TGFβ binding to a type II receptor causes the receptor to recruit and phosphorylate a type I receptor. Step 2: phosphorylated type I receptor recruits and phosphorylates Smad2 or Smad3, upon which the Smads open up and expose a dimerization surface. Step 3: phosphorylated Smad2 or Smad3 dissociates from the receptor and oligomerizes with inhibitory Smad4. Step 4: the Smad2/3–Smad4 complex migrates to the nucleus, recruits other gene regulatory proteins (blue), and activates transcription of specific target genes.

Nevertheless, important aspects of its pathogenesis have been elucidated, particularly those related to progressive fibrosis, which is one of the hallmarks of the disease. Transforming growth factor β (TGFβ) is a pivotal cytokine in this process; it is a pleiotropic cytokine that induces matrix accumulation, regulates lymphocyte function and promotes endothelial cell apoptosis. Binding of TGFβ to the type II TGFβ receptor triggers its heterodimerization with, and activation of, type I TGFβ receptor. This activation results in a downstream signaling cascade with phosphorylation of specific receptor-regulated Smad (R-Smad) proteins (Smad2/3), which partner with Smad4 after dissociation from the TGFβ receptor (Figure 1). Smad2/3–Smad4 oligomers migrate to the nucleus, recruit other gene regulatory proteins, and activate transcription of specific target genes. In the absence of ligand stimulation, Smads reside predominantly in the cytoplasm; translocation of the activated R-Smad–Smad4 complex into the nucleus is a key step in signal transduction.

Skin fibroblasts from patients with SSc express relatively high levels of TGFβ receptor, and contain high concentrations of R-Smad3 in the nucleus, while inhibitory Smad7 is functionally defective [3–5]. These and other data suggest that TGFβ signaling is constitutively activated in SSc fibroblasts, thus contributing to aberrant extracellular matrix synthesis. The important role of Smads in fibrosis is illustrated by the finding that Smad3-deficient mice are resistant to different forms of fibrosis. Not surprisingly, the TGFβ/Smad axis has been identified as a therapeutic target in fibrotic conditions such as SSc.

## A New Study in a Mouse Model of SSc

A study published in this issue of *PLoS Medicine* by Liu et al. [6] shows that, in a hybrid human SSc skin–severe combined immunodeficient mouse xenotransplant model, stabilizing microtubules using paclitaxel (Taxol; a powerful anticancer agent and angiogenic inhibitor isolated from the bark of the Pacific yew tree) reduces production of phosphorylated Smad2/3 and expression of *COL1A2* (one of the genes involved in production of collagen, whose promoter contains multiple Smad-binding elements). The end result is to lessen fibrosis histologically. The study takes advantage of an important animal model for scleroderma, the engraftment of SSc skin samples in immunodeficient mice. These samples have previously been shown to retain their phenotype and abnormal Smad expression [7]. The study also builds on previous work that has shown that microtubules provide a negative feedback loop in TGFβ signaling in cell lines by forming a complex with endogenous Smad2, Smad3, and Smad4, sequestering R-Smads away from the TGFβ receptor [8]. Taken together, these studies suggest that modulating TGFβ/Smad signaling with paclitaxel may be an effective means to treat skin fibrosis.

## The Role of Other Signaling Cascades

However, recent data indicate that other signaling cascades are also perturbed [9], and it is, therefore, conceivable that the beneficial effects of paclitaxel on scleroderma skin thickening are not solely due to changes in TGFβ/Smad signaling. One of the read-outs of fibrogenesis in the study of Liu et al. is reduced expression of *COL1A2*, an essential gene involved in the biosynthesis of collagen. However, this process is complex: extensive posttranslational modification of the *COL1A2* gene product occurs during the fibrotic process in which many key enzymes such as telopeptide lysyl hydroxylase are involved [10]. Future studies should address the effect of paclitaxel on the expression of the wide array of enzymes involved in fibrosis by genome-wide expression studies in patients treated with paclitaxel or ex vivo on scleroderma skin samples.

## Next Steps

By contrast, scleroderma-like changes in patients with cancer have been ascribed to the use of taxanes, including paclitaxel [11]. Whether, as suggested by Liu et al., this paradoxical effect on skin relates to the use of low doses in the mouse model described by them rather than the high doses used in patients with cancer remains to be determined, but the point underscores the need for further studies. Further work is also needed on the in vivo effects of paclitaxel on the vasculature and immune abnormalities in SSc patients, which are difficult to evaluate using scleroderma skin grafts in immunodeficient mice. At the low doses used in the studies by Liu et al. no antiangiogenic effect was found.

Clearly, there is a delicate balance between microtubule stabilizing and destabilizing forces in scleroderma, which paclitaxel may alter. These findings suggest, however, that a small pilot study of such therapy in selected patients with diffuse SSc, though a daring endeavor, may be worth the risk.

## Abbreviations

R-Smad: receptor-regulated Smad
SSc: systemic sclerosis
TGFβ: transforming growth factor β
